# Dual zeitgeber axes in psoriasis: a chronobiological framework for immune jet lag

**DOI:** 10.3389/fimmu.2026.1848727

**Published:** 2026-07-30

**Authors:** Haiying Lv, Guichen Ling, Huixia Mo, Minfang Lu, Danni Yao, Chuanjian Lu

**Affiliations:** 1 Second Clinical Medical College, Guangzhou University of Chinese Medicine, Guangzhou, China; 2 Department of Rheumatology, Shenzhen Traditional Chinese Medicine Hospital, Shenzhen, China; 3 Department of Chronic Disease Management, The Second Affiliated Hospital of Guangzhou University of Chinese Medicine, Guangzhou, China; 4 Department of Dermatology, The Second Affiliated Hospital of Guangzhou University of Chinese Medicine, Guangzhou, China; 5 State Key Laboratory of Traditional Chinese Medicine Syndrome, The Second Affiliated Hospital of Guangzhou University of Chinese Medicine, Guangzhou, China; 6 Guangdong–Hong Kong–Macau Joint Lab on Chinese Medicine and Immune Disease Research, Guangzhou University of Chinese Medicine, Guangzhou, China

**Keywords:** circadian rhythm, gut–skin–immune axis, immune jet lag, psoriasis, Th17, zeitgeber

## Abstract

Psoriasis is primarily characterized by interleukin-23/T helper 17 (IL-23/Th17)-related inflammation, but clinical and epidemiological observations also suggest recurrent temporal features, including seasonal fluctuation, nocturnal symptom exacerbation, sleep–wake disturbance, and circadian-related risk contexts. Here, we propose the Dual Zeitgeber Model as a hypothesis-generating and testable chronobiological framework for organizing these observations. The central hypothesis is that persistent misalignment between the light–suprachiasmatic nucleus (SCN)–neuroendocrine axis (Axis I) and the feeding–metabolism–microbiome axis (Axis II) may contribute to immune temporal desynchronization. Within this framework, immune jet lag is reserved for this hypothesized state of immune temporal desynchronization. This concept describes a condition in which neuroendocrine immune gating and metabolic–microbial immune signals may become temporally misaligned. This hypothesis raises several testable questions: whether Axis I–Axis II temporal relationships are associated with disease activity, whether abnormal immune temporal organization persists over time, and whether adjunctive circadian-oriented strategies may provide mechanistic insight or potential clinical value alongside established therapies. More broadly, this framework reframes time as a measurable, stratifiable, and testable research dimension, thereby providing new directions for circadian phenotype-based stratification, longitudinal tracking of disease trajectories and treatment responses, and the design of time-controlled intervention studies.

## 1 Introduction

Psoriasis is a chronic inflammatory skin disease jointly shaped by genetic susceptibility and environmental factors, with the IL-23/Th17 axis representing a central immune pathway (1). While biologic agents targeting the IL-23/Th17 axis substantially improve short-term lesion control and reshape the therapeutic landscape (2), real-world evidence shows that a considerable proportion of patients, during long-term follow-up, still experience loss of efficacy, reduced drug retention, and recurrent disease fluctuations (3, 4). This clinical reality, characterized by limited durability of therapeutic response and marked inter-individual variability, suggests that inflammatory intensity alone may not fully explain the complex and dynamic course of psoriasis, and that time-dependent regulatory dimensions warrant consideration within the current disease framework.

A large body of clinical and epidemiological evidence further indicates that psoriasis exhibits consistent temporal dynamic patterns. During seasonal transitions, disease activity consistently shows a pattern of exacerbation in winter and alleviation in summer (5–9). At the circadian level, symptom exacerbation is often concentrated from the evening to nighttime, forming a relatively stable circadian phenotype, accompanied by increased nocturnal pruritus and disruption of sleep architecture (10, 11). These patterns are quantitatively assessed using objective measures, making them more readily observable (12).

Epidemiological studies further suggest an association between circadian disruption and psoriasis. An analysis based on data from the National Health and Nutrition Examination Survey (NHANES) found that circadian syndrome, a composite construct incorporating insufficient sleep, depression, obesity, and hypertension, had greater predictive value for psoriasis than the traditional metabolic syndrome model. These findings suggest that circadian disruption may represent a systemic risk context relevant to psoriasis (13). Together, these time-related clinical and epidemiological observations suggest that psoriasis is not shaped solely by whether inflammatory pathways are activated, but may also involve dysregulated temporal organization of immune responses.

In this context, the skin is not merely a passive inflammatory target organ, but rather a peripheral circadian system with intrinsic circadian properties. The temporal organization of peripheral immunity may depend in part on the coordinated alignment of multiple zeitgebers. On the one hand, the light-driven central circadian network, through the hypothalamic suprachiasmatic nucleus (SCN) and its downstream neuroendocrine outputs, may provide circadian gating signals for peripheral immune responses (light–SCN–neuroendocrine axis, Axis I); on the other hand, feeding–metabolic rhythms and downstream gut microbiota-derived cues may influence peripheral immune function (feeding–metabolism–microbiome axis, Axis II). Within a broader neuroimmunological framework, the central and peripheral circadian systems are considered to communicate across both temporal and spatial dimensions, thereby contributing to the temporal organization of immune-cell distribution and function (14).

Environmental light exposure and metabolic rhythmicity are therefore plausible upstream contexts for psoriasis-related circadian regulation. Beyond seasonal variation in ultraviolet exposure, transcriptomic studies have shown that narrow-band ultraviolet B (NB–UVB), while suppressing inflammation, induces a broad circadian-related transcriptional program in lesional epidermis, involving multiple signaling networks linked to circadian regulation (15). Concurrently, systemic circadian disruption, such as shift work and chronic sleep disturbances, has been associated with an increased inflammatory burden (16). At the molecular level, experimental evidence suggests a bidirectional relationship between the circadian–regulated coagulation-related gene *F3* (tissue factor) and IL-17 signaling in keratinocytes. These findings are consistent with the possibility that local clock-related abnormalities may be associated with keratinocyte inflammatory responses in psoriasis (17).

However, existing studies have largely examined phototherapy effects, nocturnal symptomatic phenotypes, local clock-related changes, and metabolic–microbial signals in isolation. As a result, it remains difficult to determine how central light–neuroendocrine timing, peripheral feeding–metabolic–microbial timing, and local cutaneous immune responses may be conceptually integrated within a single testable framework. Drawing on the concept of peripheral circadian misalignment (18), the present review organizes these lines of evidence into the proposed Dual Zeitgeber Model. The novelty of this framework does not lie in identifying circadian disruption or immune regulation as separate phenomena; rather, it lies in positioning Axis I, Axis II, persistent Axis I–Axis II misalignment, and psoriasis-related immune temporal organization within a unified, hypothesis-generating, and testable chronobiological framework.

**Figure 1:**
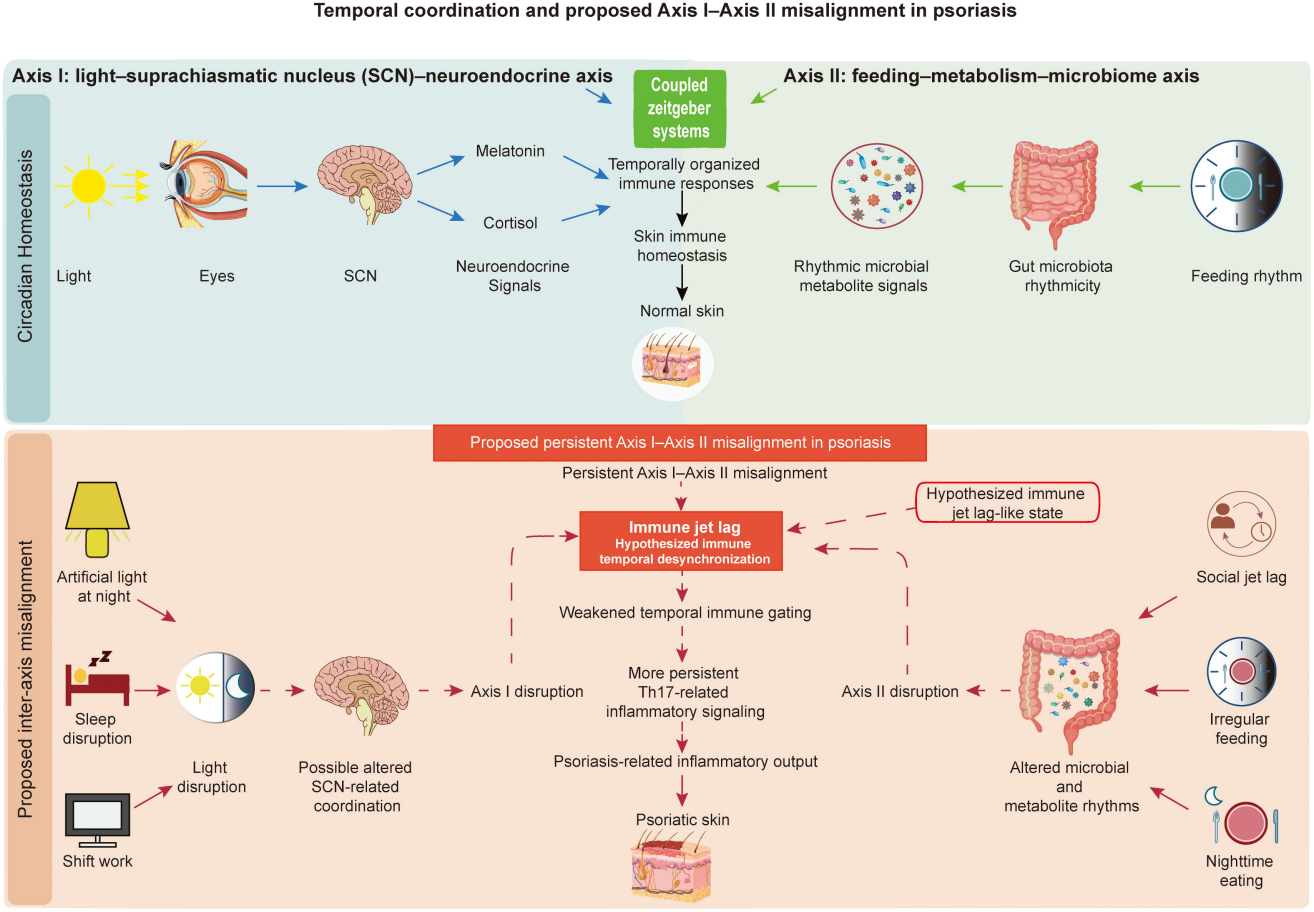
Conceptual framework of the dual zeitgeber model in psoriasis. This figure illustrates the Dual Zeitgeber Model proposed in this review. The model conceptualizes the light–SCN–neuroendocrine axis (Axis I) and the feeding–metabolism–microbiome axis (Axis II) as two coordinated yet partially dissociable zeitgeber systems. Under physiological conditions, Axis I may contribute to immune temporal organization through light–dark information, SCN-related circadian coordination, and neuroendocrine signals, whereas Axis II may link feeding–fasting rhythms, metabolic signals, gut microbiota, and microbiota-derived metabolites to peripheral immune regulation. Within the framework proposed here, persistent Axis I–Axis II misalignment is proposed as the defining condition for a hypothesized immune jet lag state, which may involve weakened temporal immune gating, more persistent Th17-related inflammatory signaling, and psoriasis-related inflammatory output. Disruption of a single axis may affect immune temporal organization, but it does not by itself define immune jet lag within the framework proposed here. This figure should be interpreted as a conceptual, hypothesis-generating, and testable chronobiological framework rather than as a validated pathogenic mechanism in psoriasis. The skin structure graphical elements were adapted from “Anatomical structure of young and old skin realistic isolated” by macrovector on Freepik. The eyeball graphical element was adapted from “Diagram of human eyeball anatomy” by brgfx on Freepik, and the intestine and gut microbiome graphical elements were adapted from “Human intestine with gut microbiome vector illustration” by brgfx on Freepik. These Freepik elements are used under the Freepik License with attribution. The brain schematic used in **Figure 1** was adapted from “Brain human sagittal section”, available through Wikimedia Commons under the Creative Commons Attribution 2.5 Generic license (CC BY 2.5; https://creativecommons.org/licenses/by/2.5/), with creative credits to Patrick J. Lynch, medical illustrator, and C. Carl Jaffe, MD, cardiologist.

Within the Dual Zeitgeber Model, the light–SCN–neuroendocrine axis (Axis I) and the feeding–metabolism–microbiome axis (Axis II) are conceptualized as coordinated yet partially dissociable zeitgeber systems. Here, immune jet lag is reserved for a hypothesized state of immune temporal desynchronization defined by persistent Axis I–Axis II misalignment. Within this framework, such misalignment may be associated with weakened temporal immune gating and a more persistent inflammatory phenotype, but this relationship remains to be directly validated in psoriasis. In the following sections, we first examine Axis I as the central circadian gating arm, then Axis II as the peripheral metabolic–microbial gating arm, and finally discuss candidate mechanistic links through which persistent Axis I–Axis II misalignment may relate to psoriasis-associated immune temporal dysregulation. This overall conceptual framework, including physiological Axis I–Axis II coordination and hypothesized pathological misalignment, is summarized in **Figure 1**.

## 2 Axis I: light–SCN–neuroendocrine axis

Axis I refers to the central light–SCN–neuroendocrine zeitgeber axis through which environmental light–dark information is transduced into rhythmic neuroendocrine outputs. Through the SCN and downstream neuroendocrine mediators such as melatonin and glucocorticoids, Axis I may provide systemic circadian gating signals that contribute to the temporal organization of immune responses (19–21). In this framework, external light exposure is viewed as an upstream zeitgeber input that may influence the timing and response thresholds of immune activity, rather than as a psoriasis-specific causal pathway in itself.

Consistent with the circadian timing context represented by Axis I, local clock-related changes in the skin may be associated with keratinocyte inflammatory responses. Recent evidence indicates that the core circadian clock protein Cryptochrome-2 (CRY2) is reduced in psoriatic lesions, and single-cell analysis further suggests reduced CRY2 expression in lesional keratinocytes. In experimental models, reduced CRY2 expression was associated with increased ERK1/2 activation, keratinocyte hyperproliferation, and inflammation-related chemokine expression, whereas the CRY2 stabilizer KL001 attenuated the corresponding phenotypes (22). These findings suggest that keratinocytes may represent a local cutaneous effector interface through which clock-related abnormalities are linked to psoriasis-related inflammatory output, thereby providing disease-relevant support for the cutaneous biological relevance of Axis I.

**Figure 2:**
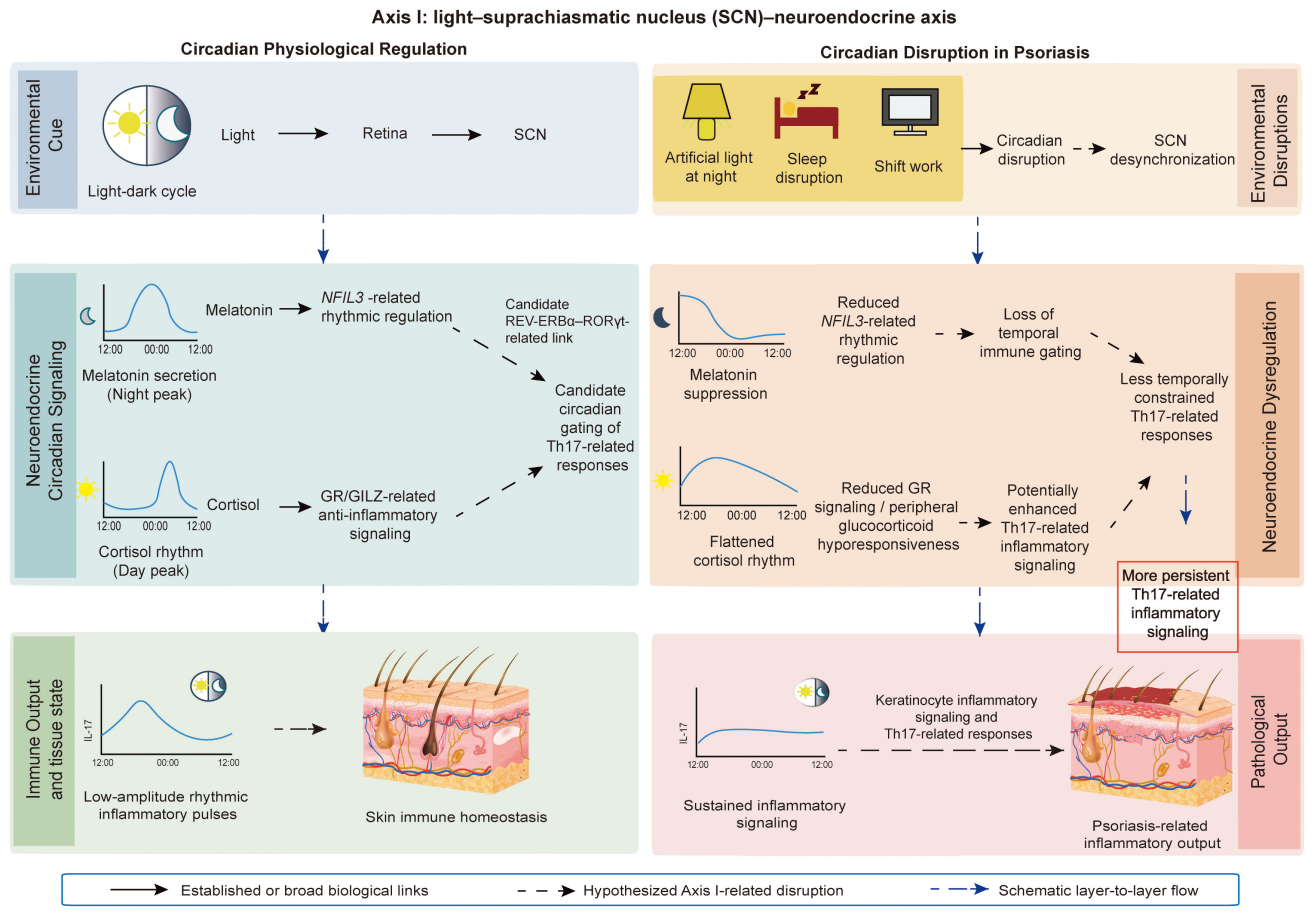
Axis I-related circadian gating and disruption context in psoriasis. This figure illustrates how the light–SCN–neuroendocrine axis (Axis I) may provide a central circadian timing context for the temporal organization of Th17-related immune responses. The left panel shows the physiological state, in which light–dark information is transmitted through the retina and SCN and is linked to rhythmic neuroendocrine outputs, including melatonin and cortisol, thereby forming candidate circadian immune-gating signals. The right panel summarizes Axis I-related disruption contexts relevant to psoriasis, including artificial light at night, sleep–wake disruption, and shift work. These factors may be associated with altered SCN-related coordination, reduced melatonin rhythmicity, flattened cortisol rhythm, and reduced peripheral glucocorticoid responsiveness. Within this hypothesized framework, Axis I-related disruption may weaken temporal immune gating, weaken temporal constraints on Th17-related responses, and be linked to more persistent inflammatory signaling and psoriasis-related inflammatory output. Curves indicate schematic rhythmic patterns rather than quantitative measurements. Solid arrows indicate established or broadly supported biological links, whereas dashed arrows indicate hypothesized links requiring direct validation. This figure should be interpreted as a hypothesis-generating conceptual framework rather than as a validated psoriasis-specific causal mechanism. The skin structure graphical elements were adapted from “Anatomical structure of young and old skin realistic isolated” by macrovector on Freepik and are used under the Freepik License with attribution.

In addition to responding to systemic circadian cues, the skin possesses autonomous peripheral circadian clocks that participate in the phase-dependent regulation of barrier homeostasis, tissue repair, and inflammatory responses (23, 24). The phase stability and amplitude of these peripheral clocks may be influenced by Axis I-related neuroendocrine zeitgeber signals, including glucocorticoids and melatonin (25–28). Under conditions of weakened Axis I-related entrainment, peripheral circadian rhythms in the skin may become more susceptible to phase shifts and amplitude reduction, potentially weakening the coordination of immune gating and repair-related programs across the day–night cycle. Together, these observations provide a conceptual basis for examining how weakened Axis I entrainment may be linked to circadian-dependent symptom fluctuation and a more persistent inflammatory phenotype in psoriasis (**Figure 2**, **Table 1**).

**Table 1:** Foundational mechanistic and psoriasis-related evidence for Axis I, the light–SCN–neuroendocrine axis.

Axis I component	Representative studies	Evidence source and study context	Key findings	Positioning within the Axis I framework
External circadian zeitgeber disruption and inflammatory consequences	Li et al., 2013 (38); Gu et al., 2024 (13); Caldarola et al., 2024 (39); Yang et al., 2025b (40); Zhang et al., 2026 (41)	Psoriasis population studies	Night-shift work, circadian-related syndromes, or sleep-related features associated with psoriasis risk, activity, or susceptibility.	Clinical upstream-disruption evidence.
	Castanon-Cervantes et al., 2010 (98)	Non-psoriasis light–dark phase-shift model	Repeated phase advances enhanced LPS-induced inflammation and endotoxin-shock mortality; central and peripheral clock-gene rhythm abnormalities.	Circadian-disruption inflammatory amplification.
	Cuesta et al., 2016 (127)	Simulated night-shift study in healthy adults	Cytokine secretion rhythms advanced after simulated night shift; selected immune-cell rhythms did not shift synchronously.	Human immune-parameter desynchronization after simulated night shift.
	Schilperoort et al., 2020 (128)	Non-psoriasis chronic inflammatory disease model	Alternating light–dark cycles aggravated atherosclerosis and increased plaque macrophage content.	Chronic-inflammation support for circadian-disruption effects.
Nocturnal neuroendocrine output: melatonin-related signaling	Mozzanica et al., 1988 (45)	Neuroendocrine observation in psoriasis	Loss of nocturnal melatonin peak; abnormal circadian melatonin rhythm.	Abnormal nocturnal melatonin-related output in psoriasis.
	Farez et al., 2015 (129)	Multiple sclerosis, autoimmune models, and T-cell experiments	MTNR1A–ERK1/2–C/EBPα–REV-ERBα/NFIL3-related melatonin signaling; reduced pathogenic Th17; promoted Tr1 responses.	Autoimmune melatonin–Th17 support.
Diurnal systemic neuroendocrine output: cortisol-related abnormalities	Repousi et al., 2022 (58)	Neuroendocrine observation in psoriasis	Flattened diurnal salivary cortisol rhythm; associations with selected psychological and disease-severity measures.	Cortisol-related diurnal abnormality in psoriasis.
	Evers et al., 2010 (56)	Longitudinal stress–cortisol observation in psoriasis	Higher daily stress levels associated with lower serum cortisol.	HPA stress-reactivity background; not a direct circadian rhythm study.
	Wright et al., 2015 (37)	Chronic circadian misalignment in healthy adults	Lower cortisol with increased TNF-α, IL-10, and CRP under chronic circadian misalignment.	Circadian misalignment effects on cortisol and inflammatory proteins.
Local cutaneous glucocorticoid-responsive anti-inflammatory buffering	Hannen et al., 2017 (57)	Psoriatic skin and related experiments	Local glucocorticoid synthesis and GR expression dysfunction in lesional and non-lesional skin.	Cutaneous glucocorticoid-responsive buffering component.
	Man et al., 2013 (59)	Psoriatic lesions, keratinocytes, and IMQ model	Impaired GR nuclear translocation; VEGF/IFN-γ-induced similar abnormalities; no cortisol or ACTH group difference at measured time points.	Local GR impairment.
	Jones et al., 2015 (61); Sevilla et al., 2019 (60)	GILZ–Th17 study and review-based support	Reduced GILZ in psoriatic lesions; stronger IMQ-induced Th17/IL-17 inflammation with GILZ deficiency.	GC/GR/GILZ–Th17 anti-inflammatory restraint.
Core clock-mediated temporal immune gating and inflammatory effector linkage	Rahman et al., 2015 (130)	Constant-routine human study and ex vivo LPS stimulation	Endogenous circadian rhythmicity of LPS-induced MCP-1, GM-CSF, IL-8, and TNF-α responses.	Endogenous temporal immune gating in humans.
	Gibbs et al., 2012 (88)	Macrophage clock and innate immune gating model	BMAL1/REV-ERBα-dependent gating of LPS-induced cytokine responses; loss of gating after circuit disruption.	Clock-controlled innate immune gating.
	Hashiramoto et al., 2010 (131)	Experimental arthritis model	*Cry1/Cry2* deficiency aggravated arthritis and increased TNF-α, IL-1β, and IL-6; anti-TNF attenuated the phenotype.	Inflammatory amplification after clock-gene disruption.
	Yu et al., 2013 (49)	Clock–Th17 regulation and inflammation model	NFIL3 repression of RORγt; REV-ERBα/NFIL3 linkage to Th17 differentiation; increased Th17 and IL-17A-dependent susceptibility after chronic light-cycle perturbation.	Foundational clock–Th17 linkage.
	Bhattarai et al., 2025 (89)	Intestinal ILC3 and infection model	REV-ERBα/β–NFIL3–RORγt circuit involved in ILC3 identity and intestinal inflammation resistance.	Clock-related immune effector programming; not psoriasis-specific.
Psoriasis-related local cutaneous clock and inflammatory effector linkage	Ando et al., 2015 (102)	*Clock/Per2* mutant IMQ model and γδ T cells	*Clock* mutation attenuated, and *Per2* mutation aggravated IMQ dermatitis; CLOCK regulated γδ T-cell IL-23R; cutaneous IL-23R showed circadian variation.	Clock-gene regulation of IL-23R-related psoriasis-like inflammation.
	Wang et al., 2021 (90)	REV-ERBα, γδT17 cells, and IMQ model	REV-ERBα deficiency increased γδT17 cells; REV-ERB agonism reduced IL-17 production and attenuated psoriasis-like dermatitis.	REV-ERB–IL-17 effector linkage.
	Greenberg et al., 2020 (42)	IMQ model, *Bmal1* deficiency, human-lesion data, and feeding-time shift	Cutaneous BMAL1 regulation of interferon-sensitive inflammation; feeding-timing effects on IMQ-induced ISG circadian patterns.	Local cutaneous clock regulation and feeding-timing interface evidence.
	Yao et al., 2026 (22)	Psoriasis samples, cell experiments, IMQ mice, and skin organoids	CRY2 downregulation linked to keratinocyte inflammation and hyperproliferation; attenuation by CRY2 stabilization.	CRY2-related keratinocyte inflammatory and proliferative effects.
	Pigatto et al., 1985 (94); Mora-Velandia et al., 2017 (95)	Immune temporal patterns and effector-cell studies in psoriasis	Circadian abnormalities in neutrophil migration; migratory and IL-17/IL-22 features of psoriasis-related ILC-like cells.	Temporal immune-migration and effector-cell background.
Inflammatory feedback on clock programs and SCN photic responsiveness	Németh et al., 2022 (87)	Psoriatic skin and keratinocyte study	Abnormal clock-gene expression in lesional and non-lesional skin; altered keratinocyte clock-gene oscillation after inflammatory stimulation.	Local inflammation–cutaneous clock interaction.
	Yuan et al., 2024 (17)	Exploratory human skin and keratinocyte study	F3 linked to IL-17/JAK–STAT-related keratinocyte inflammation; proposed bidirectional relationship with IL-17 signaling.	Exploratory inflammatory signaling–clock-associated molecule interaction; not core feedback validation.
	Cavadini et al., 2007 (96)	Synchronized NIH 3T3 fibroblasts and TNF-α-infused mice	TNF-α suppressed selected E-box-dependent clock-related genes; reduced SCN *Dbp* expression.	Cytokine feedback on clock-related molecular programs and SCN-related gene expression.
	Palomba et al., 2008 (97)	Chronic LPS-induced systemic inflammation in mice	Reduced SCN light-induced Fos responses at 1 week; no significant difference from controls at 4 weeks.	Transient inflammation-related attenuation of SCN photic responsiveness.
Clinical temporal phenotypes of psoriasis	Ferguson et al., 2021 (5); Yang et al., 2025a (9); Zheng et al., 2021 (6); Niedźwiedź et al., 2024 (8); Vlami et al., 2025 (12); Luengas-Martinez et al., 2022 (10); Liang et al., 2023 (7); Jiang et al., 2024 (11); Radaelli et al., 1982 (86)	Patients with psoriasis and related clinical data	Seasonal variation, diurnal symptom patterns, and sleep–wake-related abnormalities.	Clinical temporal phenotype basis.
Circadian-related treatment and translational observations	Addison et al., 2021 (15)	Psoriasis treatment-related observation	NB-UVB-associated epidermal transcriptional changes involving circadian-rhythm-related genes and regulators.	Circadian-related molecular programs in treatment response.
	Mohammadi et al., 2024 (50)	Topical melatonin, topical rosuvastatin, and placebo-controlled trial	Reduced mild-to-moderate plaque psoriasis severity with topical melatonin and rosuvastatin.	Preliminary topical melatonin-related translational support.
	Damiani et al., 2023 (43)	Chronomedical study in patients with psoriasis	Dietary pattern associated with MED and its circadian oscillation.	Chronomedical evidence for circadian MED variation and dietary influence.

Evidence is organized according to the functional sequence of Axis I: external circadian zeitgeber disruption, rhythmic neuroendocrine output, temporal immune gating, psoriasis-related local inflammatory effects, inflammatory feedback on clock systems, and clinical or translational observations. Non-psoriasis studies provide foundational support for specific mechanistic components and broader inflammatory biology context. These findings do not imply that the complete Axis I pathway, dual-axis misalignment, or immune jet lag has been directly validated in psoriasis. Abbreviations: ACTH, adrenocorticotropic hormone; BMAL1, brain and muscle ARNT-like 1; C/EBPα, CCAAT/enhancer-binding protein α; CLOCK, circadian locomotor output cycles kaput; CRP, C-reactive protein; CRY1/2, cryptochrome 1/2; CRY2, cryptochrome 2; E-box, enhancer box; ERK1/2, extracellular signal-regulated kinase 1/2; F3, coagulation factor III; GC, glucocorticoid; GILZ, glucocorticoid-induced leucine zipper; GM-CSF, granulocyte–macrophage colony-stimulating factor; GR, glucocorticoid receptor; HaCaT, human keratinocyte cell line; HPA, hypothalamic–pituitary–adrenal; IFN-γ, interferon-γ; IL, interleukin; IL-23R, interleukin-23 receptor; ILC-like, innate lymphoid cell-like; ILC3, group 3 innate lymphoid cell; IMQ, imiquimod; ISG, interferon-stimulated gene; JAK–STAT, Janus kinase–signal transducer and activator of transcription; LPS, lipopolysaccharide; MCP-1, monocyte chemoattractant protein-1; MED, minimal erythema dose; MTNR1A, melatonin receptor 1A; NB-UVB, narrowband ultraviolet B; NFIL3, nuclear factor interleukin 3 regulated; PER2, period circadian regulator 2; REV-ERBα/β, nuclear receptor subfamily 1 group D members 1 and 2; RORγt, retinoic acid receptor-related orphan receptor γt; SCN, suprachiasmatic nucleus; Th17, T helper 17; TNF-α, tumor necrosis factor-α; Tr1, type 1 regulatory T cell; VEGF, vascular endothelial growth factor.

### 2.1 Environmental circadian disruption and candidate mechanistic links

Modern lifestyles have profoundly altered human light exposure and sleep–wake patterns. Artificial light at night (ALAN) and shift work persistently weaken the amplitude stability and phase coherence of endogenous circadian rhythms by suppressing nocturnal melatonin secretion, disrupting sleep patterns, and altering neuroendocrine outputs (20, 29–34). Circadian misalignment among melatonin, cortisol, and inflammatory cytokines has been clinically documented in shift workers (35). Experimental sleep deprivation and circadian misalignment further lead to a mismatch between cortisol rhythms and inflammatory markers, altering cytokine balance and resulting in circadian desynchronization, which manifests as sustained low-grade chronic inflammation (36, 37).

Evidence linking circadian rhythms to psoriasis continues to accumulate. Epidemiological studies have demonstrated that shift work is significantly associated with an increased risk of psoriasis development (38), often accompanied by alterations in disease severity as well as hormonal and immunological markers (39). Significant causal associations exist between specific sleep traits and psoriasis susceptibility, exhibiting an additive effect within the context of genetic predisposition (40, 41). These findings suggest that circadian disruption may be relevant not only as a comorbid feature but also as a potential risk or persistence-related context in psoriasis, although direct causal pathways remain to be clarified.

At the clinical phenotype level, patients with psoriasis commonly exhibit sleep–wake architecture disturbances, such as sleep fragmentation and nocturnal awakenings. While such abnormalities are frequently dismissed as secondary consequences of pruritus, objective assessments based on actigraphy quantify sleep–wake architecture disturbances in patients with psoriasis, offering a promising objective indicator of impaired Axis I entrainment (12). Research suggests a bidirectional regulatory relationship between psoriasis-like skin inflammation and the circadian timing system. Skin inflammation interferes with the expression of core circadian clock genes. Conversely, the murine core clock gene brain and muscle ARNT-like 1 (*Bmal1)* confers circadian rhythmicity to skin immune responses, partly by negatively regulating interferon-sensitive genes (ISGs) such as *Irf7* (42). In phototherapy, the minimal erythema dose (MED) exhibits significant circadian variations, which are not only driven by the light–dark cycle but may also be modulated by peripheral zeitgebers such as feeding (43). These findings suggest that Axis I-related circadian disruption may alter the homeostatic threshold of cutaneous immune defense. They also support a hypothesis-generating framework for examining spatiotemporal coordination between Axis I and Axis II and their potential desynchronization in disease contexts.

### 2.2 Attenuation of nocturnal zeitgeber signals

Impaired function of Axis I is often first manifested as reduced amplitude or phase disturbance of nocturnal zeitgeber signals, that is, weakened zeitgeber signals. Melatonin, as a key nocturnal zeitgeber, not only reflects changes in light exposure but also directly participates in immune regulation and inflammation control (20, 44). Early studies suggested that patients with psoriasis exhibit a reduced amplitude of the nocturnal melatonin peak (45), indicating a potential functional deficit of this nocturnal zeitgeber in disease states. Pro-inflammatory cytokines and stress mediators reprogram pineal function, shifting melatonin synthesis from a systemic endocrine mode primarily for circadian synchronization toward a paracrine mode focused on local immunomodulation (46, 47). This shift may consequently weaken its capacity for systemic circadian synchronization. The distribution and activation of mast cells via the IgE/FcϵRI and IL-33/ST2 pathways exhibit circadian variations, while melatonin and histamine interact with core clock genes through a shared transcriptional node, nuclear factor kappa-light-chain-enhancer of activated B cells (NF-κB), to regulate the production of pro-inflammatory cytokines (48). The circadian clock inhibits retinoic acid receptor-related orphan receptor gamma t (RORγt) through the nuclear receptor subfamily 1 group D member 1(REV-ERBα)/nuclear factor interleukin 3 regulated (NFIL3) axis, thereby regulating Th17 cell differentiation. Circadian disruption relieves this inhibition, promoting Th17 responses and increasing the risk of inflammation (49). Based on these findings, we propose the following mechanistic hypothesis: in patients with psoriasis, aberrations in the light–neuroendocrine rhythm may be associated with impaired melatonin rhythmicity and reduced nocturnal peak amplitude. This attenuation may weaken NFIL3-mediated rhythmic immunoregulation, allowing IL-17-related immune responses to become less temporally constrained and potentially more persistent. Notably, topical melatonin has demonstrated positive efficacy in improving lesions in patients with mild-to-moderate psoriasis in randomized controlled trials (50). This indirectly suggests that this pathway may constitute a candidate circadian regulatory target for further investigation in disease states.

### 2.3 Compromised diurnal anti-inflammatory gating

The circadian rhythm of glucocorticoids represents one of the most critical endogenous anti-inflammatory gating mechanisms during the daytime (51–53). Under physiological conditions, cortisol typically peaks after morning awakening (54) and inhibits the expression of various pro-inflammatory cytokines through glucocorticoid receptor (GR)-mediated transcriptional regulation, thereby establishing the diurnal anti-inflammatory barrier (27, 51, 55). When the phase or amplitude of the glucocorticoid rhythm is altered, immune effectors undergo parallel circadian alterations in their functional activities.

Research suggests that patients with psoriasis may exhibit a hyporesponsive neuroendocrine phenotype, characterized by a blunted cortisol response under acute stress. This deficiency in dynamic buffering capacity is significantly correlated with the subsequent exacerbation of psoriatic lesions (56). Some studies have observed that patients with psoriasis exhibit a flattened diurnal cortisol slope or elevated allostatic load (57), which are significantly correlated with symptoms of anxiety and depression as well as disease severity (58). Notably, even in the presence of circulating glucocorticoid signals, peripheral tissues such as the skin may exhibit functional signaling decoupling, exemplified by impaired GR nuclear translocation in keratinocytes, which results in an insufficient transcriptional response of downstream anti-inflammatory genes (59).

Glucocorticoid-induced leucine zipper (GILZ), a downstream regulator of GR, restricts Th17/IL-17-associated cutaneous inflammation (60, 61), providing a mechanistic link between insufficient circadian GR signaling and overactivation of the Th17 axis. In summary, abnormalities in Axis I may not primarily manifest as a reduction in absolute hormone levels. Rather, they are more prominently characterized by reduced circadian amplitude and phase instability of zeitgeber signals, coupled with decreased peripheral responsiveness, which collectively impair the temporal gating of immune cells (62, 63).

### 2.4 Summary

Axis I may exert temporal gating on immune cells, including Th17-related immune responses, through circadian neuroendocrine outputs. Within this framework, nocturnal rhythmic inhibitory signals, represented by the melatonin–NFIL3 pathway, together with daytime anti-inflammatory gating mechanisms centered on the cortisol–GR/GILZ axis, may contribute to the temporal organization of immune responses. Chronic disruption of sleep–wake rhythms may render these temporal constraints more susceptible to amplitude reduction and phase shifts, thereby potentially diminishing circadian gating. In this context, zeitgeber signals related to feeding, metabolism, and gut microbiota may influence inflammatory responses within inappropriate circadian phase windows. Taken together, current evidence supports the regulatory relevance of Axis I across environmental circadian disruption, neuroendocrine regulation, and peripheral immune responses, while its direct causal contribution to psoriasis-specific immune temporal organization remains to be further validated (**Figure 2**).

## 3 Axis II: feeding–metabolism–microbiome axis

**Figure 3:**
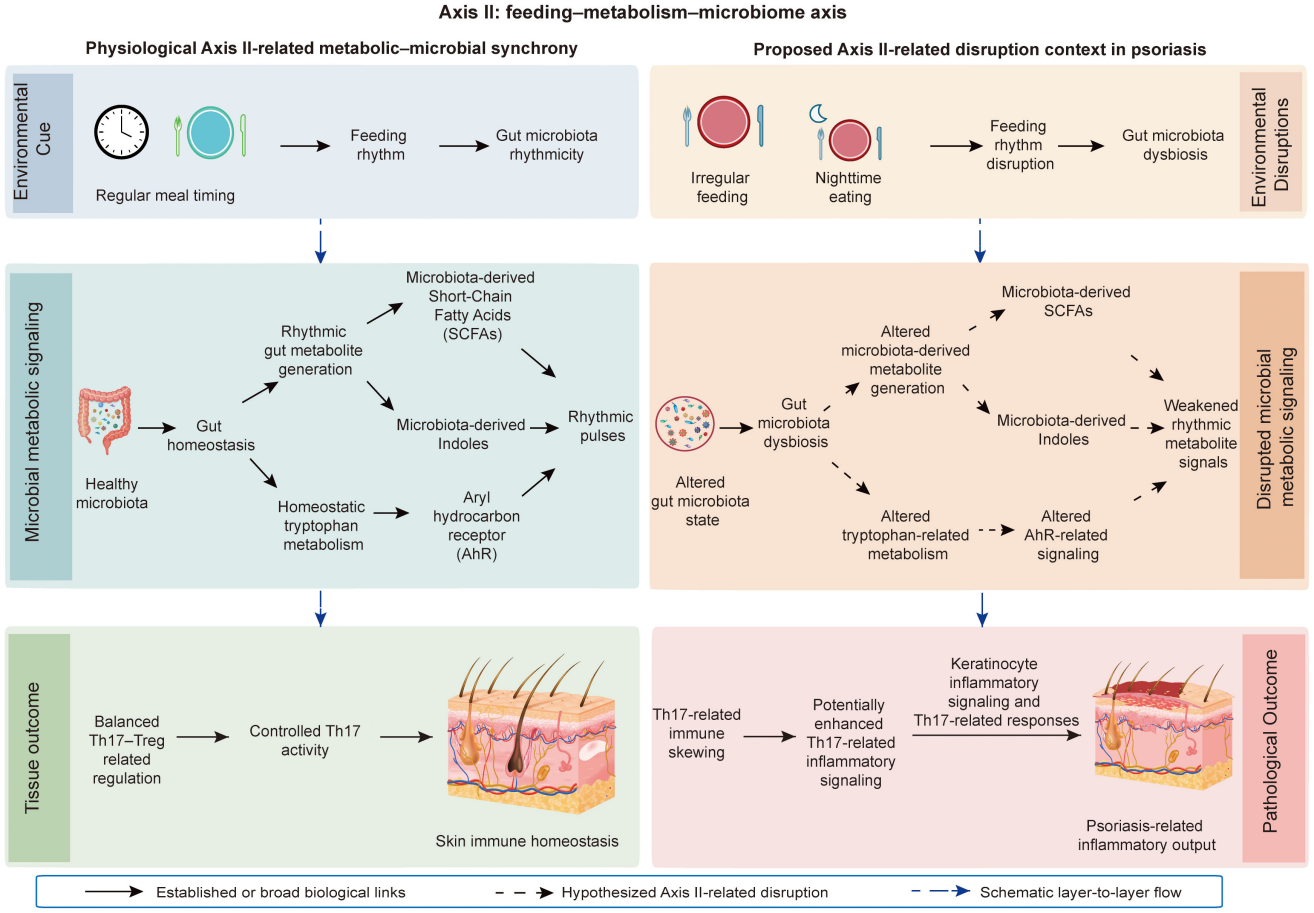
Axis II-related metabolic–microbial synchrony and disruption context in psoriasis. This figure illustrates how the feeding–metabolism–microbiome axis (Axis II) may provide a peripheral zeitgeber context linking feeding–fasting rhythms, metabolic–microbial signals, and peripheral immune regulation. The left panel shows the physiological state, in which regular feeding rhythms may be associated with gut microbiota rhythmicity, microbiota-derived metabolite generation, short-chain fatty acids, indole-related metabolites, tryptophan-related metabolism, and AhR-related signaling. These signals are presented as candidate contributors to metabolic–microbial immune regulation, Th17–Treg-related regulation, temporal constraint of Th17-related activity, and skin immune homeostasis. The right panel summarizes a proposed Axis II-related disruption context in psoriasis, including irregular feeding and nighttime eating as feeding-related disruptions. These factors may be associated with altered feeding rhythm, altered gut microbiota state, altered microbiota-derived metabolite generation, altered tryptophan-related metabolism, and altered AhR-related signaling. Within this hypothesized framework, Axis II-related disruption may weaken rhythmic metabolite signals and, through candidate metabolic–microbial immune interfaces and cutaneous effector interfaces, be linked to Th17-related immune skewing, potentially enhanced Th17-related inflammatory signaling, and psoriasis-related inflammatory output. Black solid arrows indicate established or broadly supported biological links; black dashed arrows indicate hypothesized Axis II-related links requiring direct validation; blue dashed arrows indicate schematic layer-to-layer flow. This figure should be interpreted as a hypothesis-generating conceptual framework rather than as a validated psoriasis-specific causal mechanism. The skin structure graphical elements were adapted from “Anatomical structure of young and old skin realistic isolated” by macrovector on Freepik, and the intestine and gut microbiome graphical elements were adapted from “Human intestine with gut microbiome vector illustration” by brgfx on Freepik. These elements are used under the Freepik License with attribution.

Axis I primarily represents a central light–SCN–neuroendocrine zeitgeber context, whereas peripheral clocks in tissues such as the liver, gut, and skin retain relative autonomy and can be regulated by feeding–fasting timing. Accordingly, Axis II, the feeding–metabolism–microbiome axis, refers to a peripheral zeitgeber system in which feeding–fasting rhythms are linked to peripheral immune regulation through metabolic signals, gut microbiota, and microbiota-derived metabolites (64, 65). Within this axis, nutrient-sensing pathways such as mTOR integrate feeding timing and nutrient availability into immune regulatory networks, thereby influencing CD4^+^ T-cell differentiation, particularly the Th17–Treg balance (66). At the same time, gut microbiota and microbiota-derived metabolites may also participate in metabolic–immune signaling within Axis II and may be associated with cutaneous Th17-related inflammatory responses. Within the framework proposed here, Axis II is therefore used to connect the possible relationships among feeding rhythms, metabolic–microbial signals, and the temporal organization of peripheral immunity (**Figure 3**).

### 3.1 Circadian fluctuations in microbial metabolism

Previous studies have suggested that the composition, spatial localization, and metabolic activity of the gut microbiota may exhibit circadian variation and may be influenced by host feeding timing (67–70). Microbial metabolic signals, including short-chain fatty acids (SCFAs) and indole derivatives, may also undergo temporal changes in relation to feeding–fasting rhythms and are therefore considered, within the framework proposed here, as candidate sources of peripheral temporal signals within Axis II.

Under physiological homeostasis, microbial metabolic signals such as SCFAs and indole derivatives may act on barrier sites and the peripheral immune environment in a temporally organized manner. This temporal organization may contribute to the maintenance of barrier-site homeostasis and the regulation of immune response thresholds. However, when feeding rhythms are disrupted or misaligned with the light–dark cycle, such as in social jet lag, irregular feeding, or nighttime eating, the temporal patterns of the gut microbiota and its metabolic outputs may be altered and may be accompanied by abnormalities in host metabolic or immune rhythms (69–71). SCFAs produced by microbial fermentation of dietary fiber have been implicated in the regulation of immunometabolism and T-cell differentiation, and their production may be shaped by feeding–fasting rhythms (69, 70, 72). This may in turn affect the temporal coordination between microbial metabolic signals and host immune regulation.

Gut microbiota dysbiosis and impaired intestinal barrier integrity have been reported in patients with psoriasis and have been associated with inflammation along the gut–skin axis (73–76). These alterations may weaken immune regulation and promote Th17-related inflammatory responses (77), but whether the microbiota participates in inflammatory temporal dysregulation in psoriasis requires further investigation.

### 3.2 Tryptophan–aryl hydrocarbon receptor axis: a metabolic checkpoint

Tryptophan is not only a precursor of neuroactive mediators such as serotonin but also an important source of endogenous ligands for the aryl hydrocarbon receptor (AhR), thereby providing a metabolic–immune signaling pathway through which nutrient intake, microbial metabolism, and immune activity may be linked (78, 79). In psoriasis research, microbiota-related indole metabolic signals have shown associations with cutaneous inflammation. In the *Card14*^E138A/+^ psoriasis-like mouse model, Wang et al. found that indole-producing gut microbiota promoted the production of indoxyl sulfate (I3S), and that I3S promoted psoriasis-like inflammation through AhR signaling in cutaneous Th17 cells. The study also observed that serum I3S levels in patients with psoriasis were associated with disease severity (80). By contrast, Hong et al. showed that a heat-killed *Limosilactobacillus reuteri* NCHBL-005 preparation alleviated imiquimod (IMQ)-induced psoriasis-like inflammatory skin phenotypes. Multiple AhR-active indole metabolites were detected in this preparation, and both the preparation and these metabolites modulated inflammatory responses in an M5 cytokine mixture-induced psoriasis-like HaCaT keratinocyte model (81). These findings suggest that the effects of microbiota-related indole metabolic signals on psoriasis-related inflammation may depend on the specific metabolite, target cell, and context of action. At this stage, the relevant mechanistic evidence is mainly derived from psoriasis-like animal models and cellular experiments, whereas human evidence is primarily limited to correlative observations. Representative microbial taxa or microbial functional groups reported in psoriasis-related studies, together with evidence regarding their associated metabolites, are summarized in **Supplementary Table 1**. These observations should be interpreted as evidence for microbiota-associated metabolic contexts rather than as proof that specific microorganisms are necessary or sufficient causes of psoriasis.

### 3.3 Summary

Within the framework proposed here, Axis II is used to summarize the possible links among feeding rhythms, gut microbiota, microbiota-derived metabolites, and peripheral immune responses. Current psoriasis-related evidence suggests that microbiota-related metabolic signals may be linked to cutaneous Th17-related inflammatory responses. However, whether these signals participate in the temporal organization of peripheral immunity in psoriasis in a circadian phase-dependent manner remains to be directly investigated (**Figure 3**, **Table 2**).

**Table 2:** Foundational mechanistic and psoriasis-related evidence for Axis II, the feeding–metabolism–microbiome axis.

Axis II component	Representative studies	Evidence source and study context	Key findings	Positioning within the Axis II framework
Peripheral zeitgeber basis	Damiola et al., 2000 (65)	Non-psoriasis restricted-feeding mouse study	Restricted feeding shifted peripheral circadian phase, with little change in SCN phase.	Feeding-timing input to Axis II.
Host nutrient and metabolic sensing–immune interface	Chi, 2012 (66); Delgoffe et al., 2009 (132)	Non-psoriasis immunometabolism and T-cell studies	mTOR integrates nutrient, growth factor, and leptin-related cues; mTOR deficiency impaired effector T-cell differentiation, including Th17 differentiation, and redirected activated T cells toward Foxp3^+^ Treg differentiation.	Host metabolic–immune interface.
Temporal responsiveness of gut microbiota and metabolic signals	Thaiss et al., 2016 (70); Dantas Machado et al., 2022 (133)	Non-psoriasis gut microbiota rhythmicity and feeding-pattern studies	Gut microbiota and metabolome dynamics were diurnal and responsive to diet composition and feeding patterns.	Microbial–metabolic temporal-signal layer.
Microbiota–epithelial–immune circadian homeostasis and inflammatory consequences	Tuganbaev et al., 2020 (85)	Non-psoriasis Crohn-like enteritis mouse model	Diet and feeding rhythms regulated small-intestinal microbiota–epithelial–immune homeostasis; disruption exacerbated enteritis.	Bridge to inflammatory consequences.
Feeding timing and psoriasis-related inflammatory phenotypes	Chen et al., 2023 (99); Damiani et al., 2019 (100)	Psoriasiform mouse model and observational patient study	Time-restricted feeding attenuated psoriasiform skin inflammation in mice; reduced PASI scores were observed during Ramadan fasting in patients.	Psoriasis-related feeding-timing evidence.
Human psoriasis-associated gut microbiota differences and inferred metabolic potential	Hidalgo-Cantabrana et al., 2019 (134); Xiao et al., 2021 (135)	Observational gut microbiota studies in patients with psoriasis	Gut microbiota composition differed in psoriasis; metagenomic analysis reported functional-pathway differences and inferred metabolic potential.	Human microbiota–metabolism evidence.
Human evidence of altered intestinal barrier integrity	Sikora et al., 2018 (73)	Intestinal barrier biomarker study in patients with psoriasis	Circulating claudin-3 and I-FABP levels were elevated in patients with psoriasis.	Human intestinal-barrier evidence.
I3S–AhR–skin Th17 inflammatory link	Wang et al., 2025 (80)	Psoriasiform mouse model and human correlation analysis	Indole-producing members of the gut microbiota promoted host I3S production; I3S enhanced AhR-dependent skin Th17 inflammation and correlated with disease severity.	Core microbial metabolite–Th17 link.
Strain-associated indole signals and skin inflammatory responses	Hong et al., 2026 (81)	Psoriasiform mouse and keratinocyte models	Heat-killed *Limosilactobacillus reuteri* NCHBL-005 and IAAld attenuated psoriasiform inflammation; related indole metabolites reduced keratinocyte inflammatory responses.	Context-dependent indole-signal effects.

Non-psoriasis studies provide foundational support only; current evidence does not establish the complete Axis II pathway in psoriasis, persistent Axis I–Axis II misalignment, or the full immune jet lag state. Abbreviations: AhR, aryl hydrocarbon receptor; Foxp3, forkhead box P3; I3S, indoxyl sulfate; IAAld, indole-3-acetaldehyde; I-FABP, intestinal fatty acid-binding protein; mTOR, mechanistic target of rapamycin; PASI, Psoriasis Area and Severity Index; SCN, suprachiasmatic nucleus; Th17, T helper 17; Treg, regulatory T cell.

## 4 Axis I–Axis II integration: a hypothesized mechanistic framework for immune jet lag

In the Dual Zeitgeber Model proposed in this review, the central light–dark–neuroendocrine zeitgeber context represented by Axis I and the peripheral feeding–metabolism–microbiome zeitgeber context represented by Axis II are two coordinated yet partially dissociable temporal regulatory systems. Previous studies have suggested that feeding timing can reset peripheral clocks and uncouple them from the central clock (65, 82).

Immune jet lag is defined here as a hypothetical state of immune temporal desynchronization whose defining condition is persistent Axis I–Axis II misalignment. In this state, persistent abnormalities in temporal coordination may arise between neuroendocrine-related immune gating and metabolic–microbial immune signals. Although disruption of Axis I or Axis II alone may affect the temporal organization of immune responses or inflammatory homeostasis, it does not constitute immune jet lag as defined in this review. Here, persistent Axis I–Axis II misalignment refers to disrupted temporal coordination between these two types of zeitgeber-related signals, which may involve disturbance of their original relative phase relationship even when both axes retain some degree of rhythmicity. Whether persistent Axis I–Axis II misalignment produces combined or enhanced inflammatory effects relative to single-axis disruption remains to be directly validated in psoriasis-related studies. Operational definitions of the Dual Zeitgeber Model, its two axes, immune jet lag, and Temporal Memory are summarized in **Box 1**.

Box 1 **Operational definitions of the Dual Zeitgeber Model and related concepts**

Axis I (light–suprachiasmatic nucleus [SCN]–neuroendocrine axis): A central zeitgeber axis in which the environmental light–dark cycle serves as the primary temporal cue and rhythmic neuroendocrine outputs are organized by the SCN. Through circadian variation in neuroendocrine signals, including melatonin and cortisol, this axis may participate in regulating the temporal organization of immune responses.

Axis II (feeding–metabolism–microbiome axis): A peripheral zeitgeber axis in which feeding–fasting rhythms serve as the primary temporal cue. Through metabolic signals, gut microbiota, and microbiota-derived metabolites, this axis may participate in regulating the temporal organization of peripheral immune responses.

Dual Zeitgeber Model: An integrative conceptual framework and testable chronobiological hypothesis proposed in this review. It incorporates Axis I and Axis II into a unified analytical framework to understand the potential associations between the coordination or misalignment of central and peripheral zeitgeber signals and the temporal organization of psoriasis-related immune responses. The manner in which the two zeitgeber axes interact and their specific roles in psoriasis remain to be directly validated.

Immune Jet Lag: Within the Dual Zeitgeber Model, Immune Jet Lag is proposed as a hypothetical state of immune temporal desynchronization defined by persistent Axis I–Axis II misalignment. Although disruption of Axis I or Axis II alone may affect immune rhythms or inflammatory homeostasis, it does not constitute Immune Jet Lag as defined in this review. Whether persistent Axis I–Axis II misalignment produces combined or enhanced inflammatory effects relative to single-axis disruption remains to be directly validated.

Temporal Memory: A secondary hypothetical concept proposed in this review that remains to be validated. It is intended to describe the possibility that, under conditions of prolonged circadian misalignment, immune responses may exhibit persistent shifts in temporal organization or abnormalities in circadian constraints on their timing, and may retain a tendency toward abnormal time-dependent responses even after the misalignment has been alleviated. This concept differs from conventional inflammatory memory, which primarily emphasizes enhanced response magnitude. Whether psoriasis-related immune responses exhibit the persistent temporal abnormalities described by this concept, and the molecular basis, epigenetic mechanisms, and clinical significance of such abnormalities, remain unclear.

Existing experimental studies outside the context of psoriasis provide several complementary lines of evidence relevant to the mechanistic links between these two types of zeitgeber signals. Guerrero-Vargas et al. found that the enhanced lipopolysaccharide (LPS)-induced inflammatory response observed under shift-work conditions was no longer evident when feeding during the work period was avoided, suggesting that dissociation of behavioral and feeding timing from SCN-related zeitgeber signals may alter the characteristics of immune responses (83). In mice, feeding at non-physiological times uncoupled the hepatic peripheral clock from the master SCN clock and was accompanied by worsened inflammatory outcomes following sepsis (84). Dietary composition and feeding rhythms may regulate the circadian homeostasis of the epithelial–immune barrier through the small intestinal microbiota, whereas disturbance of this temporal regulation exacerbated intestinal inflammation in mice (85). Together, these studies provide experimental support for the possibility that impaired coordination between the two axes may influence inflammatory output.

Psoriasis-related studies further provide disease-specific context and preliminary supporting observations. Clinical manifestations of psoriasis may exhibit diurnal variation (10, 86), and abnormalities in clock gene or clock protein expression have also been observed in both lesional and non-lesional skin (87). Based on these observations, the following sections discuss the potential mechanisms of immune jet lag at three levels: molecular temporal checkpoints, the local cutaneous effector interface, and inflammation–clock interactions.

### 4.1 Molecular temporal checkpoints and the temporal memory hypothesis

The circadian clock may participate in the temporal regulation of Th17 differentiation and inflammatory responses (49). REV-ERBα is an important molecular node linking the macrophage clock to inflammatory responses and may contribute to the circadian gating of certain inflammatory cytokine responses, including IL-6 (88). REV-ERB-related immune regulation has also been reported in other immune cell contexts. Bhattarai et al. showed that REV-ERBα and REV-ERBβ jointly contribute to maintaining intestinal ILC3 identity, IL-22 production, and intestinal barrier immune homeostasis through an NFIL3–RORγt-related regulatory network. Combined loss of both factors promoted the transition of ILC3s toward an ILC1-like state and increased susceptibility to inflammation associated with intestinal infection (89). These studies suggest that circadian molecular circuits may influence the temporal organization and functional state of inflammatory responses through effector nodes in different immune cell populations. A study in a psoriasis-like dermatitis model further showed that REV-ERBα deficiency was associated with increased IL-17 production by γδT17 cells, whereas the REV-ERB agonist SR9009 suppressed γδT17 responses and alleviated the IMQ-induced inflammatory skin phenotype (90). These findings suggest that REV-ERB-related molecular checkpoints may participate in the temporal regulation of psoriasis-related inflammatory responses.

Within the Dual Zeitgeber Model proposed in this review, persistent Axis I–Axis II misalignment may affect the temporal coordination among these molecular checkpoints and alter the circadian organization of IL-17-related inflammatory output. This hypothesis remains to be directly tested in psoriasis-related studies capable of simultaneously characterizing or manipulating both types of zeitgeber inputs. Building on this mechanistic background, we further propose Temporal Memory as a secondary hypothetical concept within the immune jet lag framework. This concept is intended to describe the possibility that, under conditions of persistent Axis I–Axis II misalignment, immune responses may develop persistent phase shifts or weakened circadian constraints on their timing and may retain a tendency toward abnormal time-dependent responses even after the misalignment has been alleviated. Previous studies of inflammatory memory in psoriasis have primarily focused on a propensity for recurrence-promoting responses that persists in the skin after clinical remission, potentially involving tissue-resident memory T cells, antigen-presenting cells, keratinocytes, fibroblasts, epigenetic alterations, and tissue remodeling (91). By contrast, Temporal Memory as proposed here focuses more specifically on persistent alterations in the timing of immune responses and the circadian gating of those responses. Its manifestations, persistence, molecular basis, and potential epigenetic mechanisms in psoriasis all require validation in future studies.

### 4.2 Circadian misalignment and cutaneous inflammatory amplification

In the skin, keratinocytes may represent an important candidate cutaneous effector interface through which the two types of zeitgeber-related signals influence inflammatory output and epidermal responses. Local clock-related studies have identified a bidirectional relationship between F3 and IL-17 signaling in keratinocytes (17). Reduced CRY2 expression has also been associated with keratinocyte hyperproliferation and enhanced inflammation-related responses, whereas CRY2 stabilization alleviated the corresponding phenotypes (22). Complementing these local clock-related findings, a feeding-timing intervention study further provides a bridging observation linking feeding timing to circadian immune responses in the skin. Greenberg et al. found that feeding-timing intervention altered the circadian pattern of ISG/*Irf7*-related immune responses in an IMQ-induced psoriasis-like skin inflammation model (42). These local clock-related studies suggest an association between local clock abnormalities and inflammatory or proliferative phenotypes in keratinocytes. In parallel, Greenberg et al. showed that feeding timing may affect the circadian pattern of immune responses in psoriasis-like skin inflammation.

Evidence regarding local effectors relevant to Axis II indicates that gut microbiota-derived indoxyl sulfate (I3S)–aryl hydrocarbon receptor (AhR) signaling may influence psoriasis-like inflammatory phenotypes by modulating inflammatory responses associated with cutaneous Th17 cells (80). In addition, a heat-killed *Limosilactobacillus reuteri* NCHBL-005 preparation alleviated psoriasis-like inflammatory skin phenotypes. This preparation and its AhR-active indole metabolites also modulated inflammatory responses in an M5-induced psoriasis-like HaCaT keratinocyte model (81). These studies suggest that metabolic–microbial signals may influence not only Th17-related inflammatory responses but also keratinocyte inflammatory responses.

Local inflammatory amplification may also involve the temporally patterned recruitment and cutaneous localization of inflammatory cells. Leukocyte recruitment does not occur continuously at a uniform rate. Scheiermann et al. found that leukocyte recruitment to tissues is regulated by circadian rhythms and local sympathetic neural signals. In the bone marrow and skeletal muscle systems examined in their study, experimental light–dark misalignment disrupted the corresponding tissue-specific recruitment rhythms (92). Druzd et al. further showed that lymphocyte entry into and egress from lymph nodes are also subject to circadian regulation (93). In patients with psoriasis, neutrophil migration exhibited a marked circadian rhythm that was not observed in healthy controls (94). In addition, a psoriasis-related study identified an ILC-like cell population with migration-associated features, providing a disease-related clue to the potential involvement of migratory immune effector cells in cutaneous inflammation (95). Taken together, these temporal migration data and psoriasis-related cellular observations suggest that the temporal pattern of inflammatory cell entry into or localization within the skin may constitute another potential effector component of local inflammatory amplification. Whether this temporal pattern is influenced by persistent Axis I–Axis II misalignment remains to be validated.

Based on these observations, persistent Axis I–Axis II misalignment may contribute to abnormal and persistent local inflammatory output in the skin. Keratinocytes, Th17-related responses, and temporally patterned inflammatory-cell recruitment and localization may represent candidate effector components of this cutaneous manifestation of immune jet lag. Future studies should examine the phase-dependent responses of these cutaneous effector components in time-controlled models of psoriasis-like inflammation and further test this hypothesis in conjunction with circadian phenotyping in patients.

### 4.3 Inflammatory-clock interactions and temporal dysregulation

The local inflammatory environment may, in turn, affect the cutaneous temporal regulatory network. In human psoriatic skin samples, Németh et al. observed disrupted expression of clock genes in both lesional and non-lesional areas, including reduced expression of *rev-erbα*. In inflammation-stimulated HaCaT keratinocytes, TNF-α also altered the oscillation amplitudes of clock genes, including *bmal1*, *cry1*, *rev-erbα*, and *per1* (87). At the level of transcriptional regulation, Cavadini et al. further showed that TNF-α suppressed the expression of selected period circadian regulator 1 (*Per*) genes and PAR bZip clock-controlled genes by interfering with CLOCK–BMAL1-mediated E-box-dependent transcription (96). In psoriasis-related experimental studies, the bidirectional relationship between F3 and IL-17 signaling in keratinocytes provides additional local disease-related support for this possibility (17). Taken together, these findings suggest that cutaneous inflammatory signals and abnormalities in local clock-related molecules may influence each other.

The influence of inflammation on temporal regulatory networks may not be confined to the skin. A study in mice outside the context of psoriasis suggested that chronic inflammation may transiently alter SCN responses to photic stimulation (97). This finding provides analogous support for the possibility that inflammation may feed back on Axis I entrainment responses; however, whether this occurs in psoriasis and contributes to persistent Axis I–Axis II misalignment remains to be directly validated. Conversely, abnormalities in temporal regulation may alter the propensity for inflammatory responses. In mice, chronic jet lag enhanced innate immune responses to LPS and was accompanied by altered clock rhythms in macrophages (98). Thus, within the framework proposed here, persistent inflammation and abnormalities in temporal coordination may form a mutually reinforcing process of inflammation and temporal dysregulation. Inflammatory signals may further disrupt local and central temporal regulatory components, whereas persistent Axis I–Axis II misalignment may make inflammatory output less likely to return to a normal pattern of circadian organization. This potentially reinforcing process may represent one mechanistic avenue for explaining the persistence of immune jet lag; however, its manifestations in psoriasis, its key cellular components, and its relationship to recoordination of the two axes require further investigation.

## 5 Clinical translation and therapeutic implications

Based on the dual zeitgeber model proposed in this review, circadian misalignment and abnormal temporal organization of immune responses may provide an additional perspective for understanding disease fluctuations and variability in treatment responses. However, this framework does not imply that the goals of psoriasis treatment should shift from controlling inflammation to achieving immune resynchronization. Approaches aimed at immune resynchronization are more appropriately framed as potential complementary strategies to be investigated alongside established psoriasis therapies, and their clinical value remains to be validated. This framework also does not imply that effective intervention would necessarily require simultaneous targeting of both axes; whether axis-specific or combined circadian-oriented approaches provide distinct value remains a question for future studies. Further research should evaluate whether such strategies provide additional value with respect to long-term disease control, relapse risk, treatment durability, and patient stratification. At this stage, examining psoriasis management through the lens of circadian regulation and research on treatment timing primarily serves to identify testable clinical research directions.

### 5.1 Lifestyle intervention

Lifestyle interventions, such as time-restricted feeding (TRF), warrant investigation as potential non-pharmacological approaches relevant to immune resynchronization. A two-week TRF regimen effectively alleviates psoriasis-like inflammation by modulating the proportion of Treg cells in the skin (99). Furthermore, gut-derived metabolites have been demonstrated to directly drive pathogenic Th17 cells in the skin, providing mechanistic evidence for how feeding rhythm disruption amplifies inflammatory responses across tissue compartments (80). These findings support further investigation of whether TRF may influence peripheral circadian rhythms and the rhythmic exposure pattern of metabolic cues relevant to psoriasis-related inflammation. Consistent with these findings, Ramadan fasting has also been associated with a significant reduction in Psoriasis Area and Severity Index (PASI) scores among psoriasis patients (100). Future studies could examine whether combining TRF with sleep–wake management influences temporal coupling between gut–immune regulation and cutaneous inflammatory responses within the Dual Zeitgeber Model. However, any future clinical translation of such interventions would require clearly defined safety boundaries. For specific populations at risk of malnutrition, underweight, or eating disorders, these strategies should only be implemented under strict medical supervision (100, 101).

### 5.2 Treatment timing and chronotherapeutic considerations

The diurnal fluctuations in psoriasis symptoms, together with experimental evidence linking core circadian regulators to IL-23/Th17-related inflammatory responses, provide a biological rationale for investigating treatment timing in psoriasis. Experimental studies show that *Clock/Bmal1* and their downstream circadian factors directly affect the intensity and circadian distribution of psoriasis-like inflammation (102). Epigenetic studies suggest that circadian clock proteins REV-ERBα/REV-ERBβ regulate RORγt expression and participate in maintaining the circadian stability of immune cell function (89). Melatonin is not only a nighttime output signal of Axis I, but may also participate in the recoupling of the peripheral circadian system by regulating mucosal immunity, gut microbiota metabolism, and the circadian timing system (103–105). Accordingly, future research should evaluate whether treatment timing, informed by circadian phase and pharmacokinetic profiles, influences therapeutic outcomes or endogenous circadian rhythms.

In this context, randomized controlled trials have indicated that topical melatonin improves skin lesions in patients with mild-to-moderate plaque psoriasis (50). Furthermore, 311 nm narrowband ultraviolet B (NB-UVB) activates cutaneous signaling pathways associated with circadian rhythms, redox homeostasis, and apoptosis (15). Together with evidence that cutaneous DNA repair capacity is phase-dependent (106, 107), these findings provide a rationale for further investigation of treatment timing in phototherapy.

### 5.3 Metabolic–immune signals and safety considerations

Among candidate intervention-related directions relevant to Axis II, AhR is one of the metabolic–immune regulatory nodes for which clinical intervention evidence in psoriasis is currently available. Topical tapinarof, an AhR-targeting agent, has demonstrated efficacy in clinical studies in adults with plaque psoriasis, suggesting that AhR is clinically targetable (108, 109). However, the available clinical findings for tapinarof support the efficacy of topical AhR-targeted treatment in psoriasis, but are insufficient to infer that tapinarof exerts circadian or immune resynchronization effects. Given the roles of AhR in skin barrier function, epidermal differentiation, and immune regulation mediated by ligands derived from diet, commensal microbiota, and metabolism (110–112), AhR may, within the Dual Zeitgeber Model proposed in this review, be considered a candidate mechanistic node linking metabolic signals, immune regulation, and cutaneous inflammatory responses. It may also provide an entry point for further investigation of other metabolism-related signals in Axis II-related intervention research.

AMP-activated protein kinase (AMPK) agonists, such as metformin, have been shown to suppress inflammasome activation by mimicking fasting-like metabolic signals (113). Phase-dependent interventions targeting SCFAs, such as butyrate (114–116), further suggest that the immune effects of peripheral metabolic signals depend not only on their composition but also on their temporal structure. Bile acids, as another class of pivotal metabolic zeitgebers, have been linked to mucosal barrier integrity, defense responses, and circadian regulation (117, 118). However, because clinical evidence in psoriasis stems primarily from observational studies, these pathways currently remain more suitable as prospective research directions rather than immediate therapeutic strategies (119).

It must be emphasized that clinical practice should remain vigilant against the potential impact of iatrogenic desynchronization on long-term prognosis. In chronic inflammatory states, the body often exhibits insufficient endogenous cortisol secretion relative to the inflammatory load (120–122). In this context, prolonged and disordered glucocorticoid intervention may disrupt endogenous cortisol homeostasis through a negative feedback mechanism, impairing the dynamic regulatory capacity of the hypothalamic–pituitary–adrenal (HPA) axis, thereby exacerbating circadian desynchronization (123, 124) and increasing the risk of inflammation−associated steroid resistance (121). Furthermore, non-physiological glucocorticoid exposure not only disrupts the circadian fluctuations of cortisol but also shifts the expression phases of core clock genes (such as *CLOCK, BMAL1*, and *PER3*) within peripheral immune cells and adipose tissues. These findings suggest that inappropriate steroid administration may compromise circadian stability at the tissue level (125, 126).

Taken together, these considerations support further investigation of rhythm-oriented approaches, including optimization of treatment timing, as potential complementary strategies alongside established psoriasis therapies. Future longitudinal cohort studies and time-controlled intervention studies are needed to assess whether such strategies provide additional value with respect to treatment durability, treatment switching, and patient stratification.

## 6 Limitations and future directions

Although the immune jet lag model proposed in this review provides a novel perspective for understanding psoriasis, it still faces challenges in clinical translation and mechanistic depth. First, regarding the universality and hierarchical logic of the model, although it centers on psoriasis, this framework requires extensive validation in other chronic inflammatory diseases characterized by pronounced circadian features. Simultaneously, the causal weighting of Axis I versus Axis II in disease progression remains elusive. There is an urgent need for dynamic longitudinal studies to clarify the temporal relationship between central drivers and peripheral amplification.

Second, regarding the dearth of high-resolution clinical evidence, the field currently lacks systematic longitudinal data. Future research should focus on constructing multidimensional integrated monitoring systems to evaluate the intrinsic correlations between intraday PASI score fluctuations, localized clock gene expression in psoriatic lesions, and oscillations of gut-derived metabolites. Furthermore, a critical bottleneck in developing precision chronomedicine interventions lies in mechanistically distinguishing specific inflammation induced by temporal misalignment from non−specific inflammatory responses triggered by obesity or sleep deprivation.

Finally, regarding the epigenetic nature of temporal memory, it is important to elucidate how the immune system establishes pathological homeostasis through epigenetic remodeling. Whether the proposed temporal regulatory mechanisms could inform future complementary strategies alongside established psoriasis therapies remains to be directly validated.

## 7 Conclusion

Within the proposed framework, immune jet lag refers to a hypothesized state in which Axis I and Axis II remain persistently misaligned, such that neuroendocrine immune gating and metabolic–microbial immune signals may no longer converge within appropriate circadian phase windows. In this state, temporal dysregulation of circadian immune checkpoints and metabolic–immune sensors, including NFIL3, REV-ERBα, and AhR, may contribute to more persistent inflammatory signaling rather than phase-constrained immune regulation. Therefore, the proposed framework may provide a complementary perspective for exploring circadian-informed research directions alongside established anti-inflammatory and biologic therapies. Future longitudinal multi-omics studies and time-controlled clinical trials are needed to test whether immune resynchronization-oriented strategies can provide additional value for treatment durability, relapse-related outcomes, and patient stratification.

## Data Availability

The original contributions presented in the study are included in the article/**Supplementary Material**. Further inquiries can be directed to the corresponding authors.
