# Supplementary material for: Dual zeitgeber axes in psoriasis: a chronobiological framework for immune jet lag
Source: Front Immunol. 2026 Jul 30;17:1848727. doi: 10.3389/fimmu.2026.1848727 (PMC13469100; doi:10.3389/fimmu.2026.1848727)
Supplement: Supplementary file 1 [file Table1.docx]

**Supplementary Table 1.** Representative microbial taxa, metabolic signals, and temporal features related to the feeding–metabolism–microbiome axis

| Axis II-related evidence component | Representative studies | Evidence source and study context | Representative microbial taxa or functional groups | Associated metabolic signals or temporal features | Relevance to the Axis II framework |
| --- | --- | --- | --- | --- | --- |
| Diurnal variation and feeding responsiveness of gut microbiota and metabolic signals | Leone et al., 2015(1);  Thaiss et al., 2016(2);  Dantas Machado et al., 2022(3) | Non-psoriasis contextual evidence: mouse circadian rhythmicity and feeding-intervention studies | Lachnospiraceae-related taxa, *Oscillospira*, *Lactococcus*, *Lactobacillus reuteri*, *Bacteroides acidifaciens*, *Mucispirillum schaedleri* | SCFAs, H₂S, microbial metabolome profiles, bile acid pool, FXR signaling, GLP-1 regulation | Feeding and circadian responsiveness of gut microbiota and microbiota-related metabolic signals |
| Human psoriasis-associated gut microbiota and predicted or inferred functional and metabolic features | Hidalgo-Cantabrana et al., 2019(4);  Chen et al., 2018(5);  Xiao et al., 2021(6) | Psoriasis population evidence: observational gut microbiota studies | *Ruminococcus*, *Bifidobacterium*, *Bacteroides*, *Prevotella*, *Paraprevotella*, *Alistipes*, *Faecalibacterium*; inconsistent directions across cohorts | Predicted or inferred carbohydrate transport and metabolism, bacterial chemotaxis, cobalamin and iron transport, H₂S, isovalerate, isobutyrate, hyaluronan, hemicellulose-related features | Human psoriasis-associated gut microbiota differences and inferred functional and metabolic features |
| Diet-related gut microbiota changes and psoriasis-like inflammation | Sonomoto et al., 2023(7) | Psoriasis-like model evidence: high-fat diet-associated psoriasis-like mouse model | High-fat diet-associated gut microbiota changes; *Akkermansia muciniphila* | No specific secondary metabolite linked to *A. muciniphila* | High-fat diet-associated gut microbiota changes linked to IL-17-related psoriasis-like inflammation |
| Indole-producing gut microbial groups, I3S, and AhR-mediated Th17 inflammation | Wang et al., 2025(8) | Psoriasis-like model and patient-associated evidence: *Card14*^E138A/+^ mouse model and patient correlation analysis | Indole-producing gut microbial groups | Indoxyl sulfate (I3S) | Indole-producing gut microbial groups, host I3S biosynthesis, and AhR-associated skin Th17 inflammation |
| *L. reuteri*-associated indole metabolites and AhR-associated IL-17A inflammatory regulation | Hong et al., 2026(9);  Meng et al., 2026(10) | Psoriasis-like model evidence: IMQ mouse models and microbiota-derived indole metabolite studies | *Limosilactobacillus reuteri* NCHBL-005; *Lactobacillus reuteri* DSM17938 | NCHBL-005: IAAld, I3C, ILA; DSM17938: ILA | *L. reuteri*-associated indole metabolites and AhR-associated IL-17A-related inflammatory regulation |
| Gut microbiota-related fatty acid changes and psoriasis-like inflammation | Zhao et al., 2023(11) | Psoriasis-like model evidence: K14-VEGF and IMQ mouse models with fatty acid intervention | Increased *Prevotella*; reduced *Parabacteroides distasonis* | Increased oleic acid and stearic acid | Gut microbiota-associated fatty acid changes linked to psoriasis-like inflammation |
| Gut microbiota-related bile acid changes and metabolic abnormalities | Hao et al., 2022(12) | Psoriasis-like model evidence: K14-VEGF mouse model with metabolic-disorder outcomes | Reduced *Parabacteroides distasonis* | Reduced fecal CDCA | Gut microbiota-associated bile acid changes and metabolic abnormalities |
| Specific bacterial species associated with psoriasis-like inflammation | Okada et al., 2020(13);  Chen et al., 2021(14) | Psoriasis-like model evidence: IMQ mouse models with bacterial administration or supplementation | *Staphylococcus aureus*, *Streptococcus danieliae*, *Lactobacillus reuteri* | No specific corresponding metabolites reported | Specific bacterial species associated with exacerbated or attenuated psoriasis-like inflammation |
| Note: Non-psoriasis mouse studies are included only to illustrate feeding and circadian responsiveness of gut microbiota and related metabolic signals; they do not indicate that rhythmic abnormalities of specific microbial taxa or metabolites have been validated in psoriasis. Human observational studies support psoriasis-associated gut microbiota differences and predicted or inferred functional and metabolic features, but do not support causal attribution. Findings from psoriasis-like animal models should not be directly extrapolated as evidence that any microbial taxon is necessary or sufficient for psoriasis pathogenesis, that any microbial taxon has a causative or protective role in human psoriasis, or that a causal gut microbiota–metabolite mechanism has been validated in human psoriasis. Abbreviations: AhR, aryl hydrocarbon receptor; CDCA, chenodeoxycholic acid; FXR, farnesoid X receptor; GLP-1, glucagon-like peptide-1; H₂S, hydrogen sulfide; I3C, indole-3-carbinol; I3S, indoxyl sulfate; IAAld, indole-3-acetaldehyde; ILA, indole-3-lactic acid; IMQ, imiquimod; SCFAs, short-chain fatty acids. | | | | | |

**References**

1.	Leone V, Gibbons SM, Martinez K, Hutchison AL, Huang EY, Cham CM, Pierre JF, Heneghan AF, Nadimpalli A, Hubert N, et al. Effects of diurnal variation of gut microbes and high-fat feeding on host circadian clock function and metabolism. *Cell Host Microbe* (2015) 17:681–689. doi: 10.1016/j.chom.2015.03.006

2.	Thaiss CA, Levy M, Korem T, Dohnalová L, Shapiro H, Jaitin DA, David E, Winter DR, Gury-BenAri M, Tatirovsky E, et al. Microbiota Diurnal Rhythmicity Programs Host Transcriptome Oscillations. *Cell* (2016) 167:1495-1510.e12. doi: 10.1016/j.cell.2016.11.003

3.	Dantas Machado AC, Brown SD, Lingaraju A, Sivaganesh V, Martino C, Chaix A, Zhao P, Pinto AFM, Chang MW, Richter RA, et al. Diet and feeding pattern modulate diurnal dynamics of the ileal microbiome and transcriptome. *Cell Rep* (2022) 40:111008. doi: 10.1016/j.celrep.2022.111008

4.	Hidalgo-Cantabrana C, Gómez J, Delgado S, Requena-López S, Queiro-Silva R, Margolles A, Coto E, Sánchez B, Coto-Segura P. Gut microbiota dysbiosis in a cohort of patients with psoriasis. *Br J Dermatol* (2019) 181:1287–1295. doi: 10.1111/bjd.17931

5.	Chen Y-J, Ho HJ, Tseng C-H, Lai Z-L, Shieh J-J, Wu C-Y. Intestinal microbiota profiling and predicted metabolic dysregulation in psoriasis patients. *Exp Dermatol* (2018) 27:1336–1343. doi: 10.1111/exd.13786

6.	Xiao S, Zhang G, Jiang C, Liu X, Wang X, Li Y, Cheng M, Lv H, Xian F, Guo X, et al. Deciphering Gut Microbiota Dysbiosis and Corresponding Genetic and Metabolic Dysregulation in Psoriasis Patients Using Metagenomics Sequencing. *Front Cell Infect Microbiol* (2021) 11:605825. doi: 10.3389/fcimb.2021.605825

7.	Sonomoto K, Song R, Eriksson D, Hahn AM, Meng X, Lyu P, Cao S, Liu N, Taudte RV, Wirtz S, et al. High-fat-diet-associated intestinal microbiota exacerbates psoriasis-like inflammation by enhancing systemic γδ T cell IL-17 production. *Cell Rep* (2023) 42:112713. doi: 10.1016/j.celrep.2023.112713

8.	Wang C, Dai S, Zhang S, Zheng Z, Zhou Z, Chen Z, Wang M, Gao Y, Xin Y, Xiong W, et al. Gut microbe-derived metabolites drive psoriatic inflammation via modulation of skin Th17 cells. *Immunity* (2025) 58:2241-2255.e7. doi: 10.1016/j.immuni.2025.08.006

9.	Hong E-H, Hyeong J, Ahn J-H, Han Y, Kim S, Kim SM, Yoo HH, Jeong H, Cho H-J, Park J-H, et al. Limosilactobacillus reuteri alleviates psoriasis via aryl hydrocarbon receptor-mediated regulation of Interkeukin-17A. *Int Immunopharmacol* (2026) 172:116194. doi: 10.1016/j.intimp.2026.116194

10.	Meng Z, Ren J, Lai Y, Lu S, Wu H, Jiang Y, Zhang Z, Tan G, Shi Z. Indole-3-Lactic Acid Attenuates IMQ-Induced Psoriasiform Dermatitis in Mice via AhR-Dependent Suppression of IL-17A. *Psoriasis (Auckl)* (2026) 16:585731. doi: 10.2147/PTT.S585731

11.	Zhao Q, Yu J, Zhou H, Wang X, Zhang C, Hu J, Hu Y, Zheng H, Zeng F, Yue C, et al. Intestinal dysbiosis exacerbates the pathogenesis of psoriasis-like phenotype through changes in fatty acid metabolism. *Signal Transduct Target Ther* (2023) 8:40. doi: 10.1038/s41392-022-01219-0

12.	Hao Y, Zhou P, Zhu Y-J, Zou S, Zhao Q, Yu J, Hu Y, Li J. Gut Microbiota Dysbiosis and Altered Bile Acid Catabolism Lead to Metabolic Disorder in Psoriasis Mice. *Front Microbiol* (2022) 13:853566. doi: 10.3389/fmicb.2022.853566

13.	Okada K, Matsushima Y, Mizutani K, Yamanaka K. The Role of Gut Microbiome in Psoriasis: Oral Administration of Staphylococcus aureus and Streptococcus danieliae Exacerbates Skin Inflammation of Imiquimod-Induced Psoriasis-Like Dermatitis. *Int J Mol Sci* (2020) 21:3303. doi: 10.3390/ijms21093303

14.	Chen H-L, Zeng Y-B, Zhang Z-Y, Kong C-Y, Zhang S-L, Li Z-M, Huang J-T, Xu Y-Y, Mao Y-Q, Cai P-R, et al. Gut and Cutaneous Microbiome Featuring Abundance of Lactobacillus reuteri Protected Against Psoriasis-Like Inflammation in Mice. *J Inflamm Res* (2021) 14:6175–6190. doi: 10.2147/JIR.S337031
